# Mind the gap: life-threatening retropharyngeal haematoma resulting in acute airway obstruction following blunt trauma by closing train carriage doors

**DOI:** 10.1259/bjrcr.20200190

**Published:** 2021-04-21

**Authors:** Shaneil V Patel, Abbas Reza, Scott R Rice

**Affiliations:** 1 Speciality Trainee in Clinical Radiology, East of England Imaging Academy, The Cotman Centre, Norfolk & Norwich University Hospitals NHS Foundation Trust, Norwich, UK; 2 Speciality Trainee in Clinical Radiology, Northwick Park Hospital, London North West University Healthcare NHS Trust, London, UK; 3 Consultant Interventional Head & Neck Radiologist, Northwick Park Hospital, London North West University Healthcare NHS Trust, London, UK

## Abstract

Delayed life-threatening airway obstruction due to venous injury following blunt, non-penetrative trauma to the neck.

A rare case of rapid force, blunt trauma by closing train carriage doors, leading to injury to the left internal jugular vein, subsequent retropharyngeal haematoma and airway obstruction. There was a significant delay of a few hours between injury and acute deterioration. Initial dual phase CT (unenhanced and arterial) studies identified the large retropharyngeal haematoma but the assessment of the source was inconclusive likely due to the venous injury becoming compressed by the swelling/haematoma at the time of investigation. Subsequent triple phase (unenhanced, arterial and venous) studies were performed identifying a flap in the left internal jugular vein as the likely site of vascular injury. A venous origin of haemorrhage supported the patients delayed onset of symptoms following the injury.

We suggest with blunt force trauma to the neck, in the context of suspicion of haematoma and airway compromise, the radiologist should consider protocolling a triple phase (unenhanced, arterial and venous) angiographic study.

## Clinical presentation

An 83-year-old male presented to the emergency department (ED) of a London teaching hospital with increasing and progressive difficulty in breathing and swallowing. Two hours prior to this, he sustained accidental blunt force trauma to his neck, having been caught by the closing doors of a London Underground *Tube* carriage. At the time of the incident, he reported only mild left neck pain, proportionate to the injury, but was otherwise asymptomatic. He carried on his tube journey without concern. However, after 60 min he developed worsening neck pain and decided to seek medical attention.

He had no significant medical history other than type 2 diabetes and well-controlled hypertension

Whilst travelling to the ED, approximately 90 min following the initial injury, he noted progressive inability to swallow his saliva. On arrival he had dysphagia, was repeatedly expectorating and had mild stridor. No significant neck swelling was identified at triage.

Within 10 min of arrival to the ED, he was observed to develop rapidly progressive, bilateral swelling of the neck and signs of acute airway compromise including marked stridor, voice change, respiratory distress and acute agitation.

The patient was immediately transferred to the resuscitation area of the ED and Anaesthetic and ENT support was requested urgently.

Immediate treatment included placement of a nasopharyngeal airway and high-flow (15L) oxygen via a non-rebreathable bag. The patient maintained oxygen saturations (SpO2) of <96% throughout and did not lose consciousness.

Following joint ENT and anaesthetic review, the patient was intubated using an awake fibre-optic technique and rapid sequence induction. Direct visualisation of the upper aero-digestive tract demonstrated diffuse mucosal oedema and copious secretions. No haemorrhage or mucosal injury was identified.

## Investigations

Initial haematological results demonstrated microcytic anaemia (Hb: 95 g l^−1^). Historical data documented a baseline of 126 g l^−1^.

Immediate contrast-enhanced dual phase (unenhanced and angiographic) CT (CTA) of the head and neck was performed with satisfactory opacification extracranial arterial tree.

**Figure 1.**
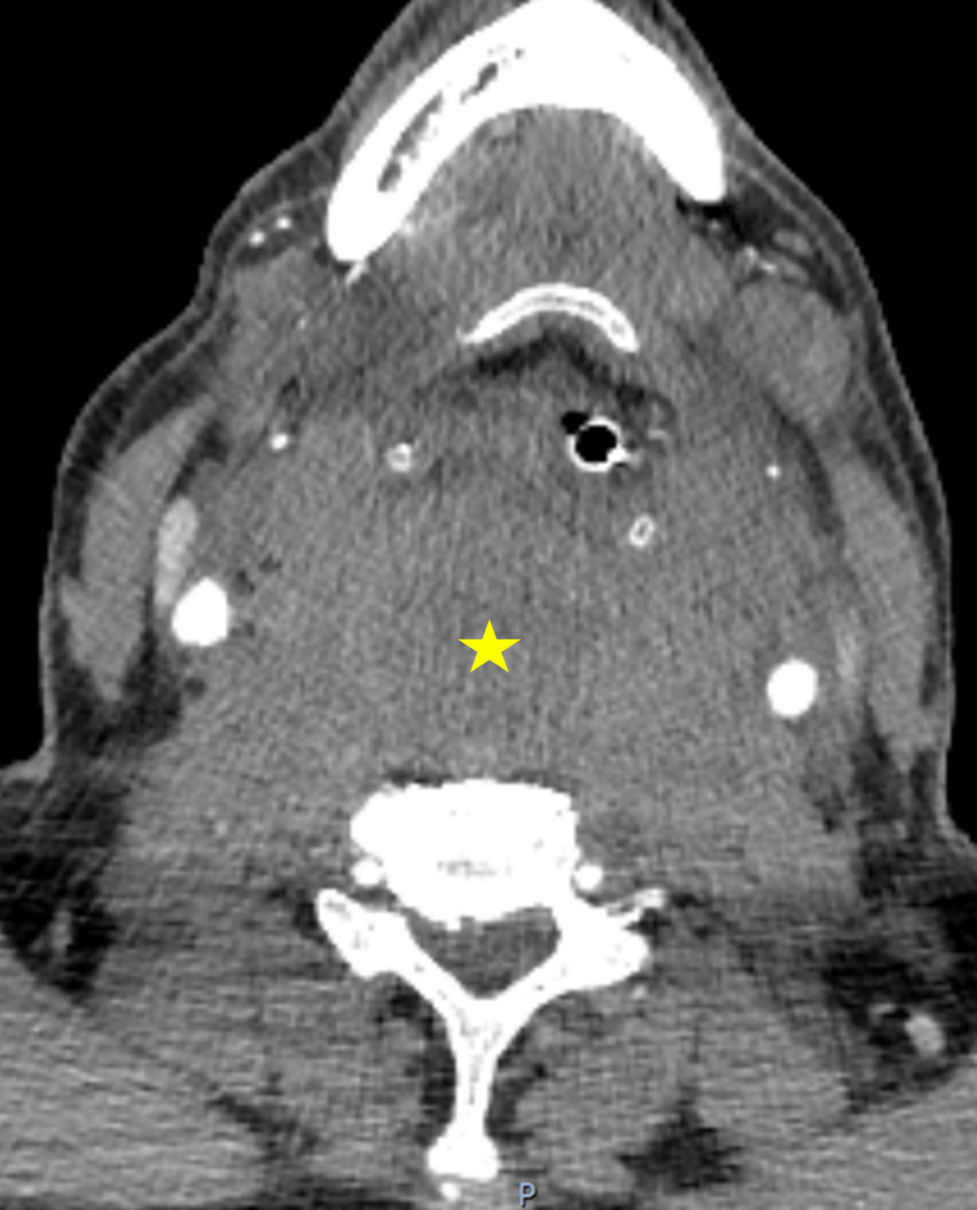
Extensive retropharyngeal haematoma effacing the larynx and supraglottic airway anteriorly.

**Figure 2.**
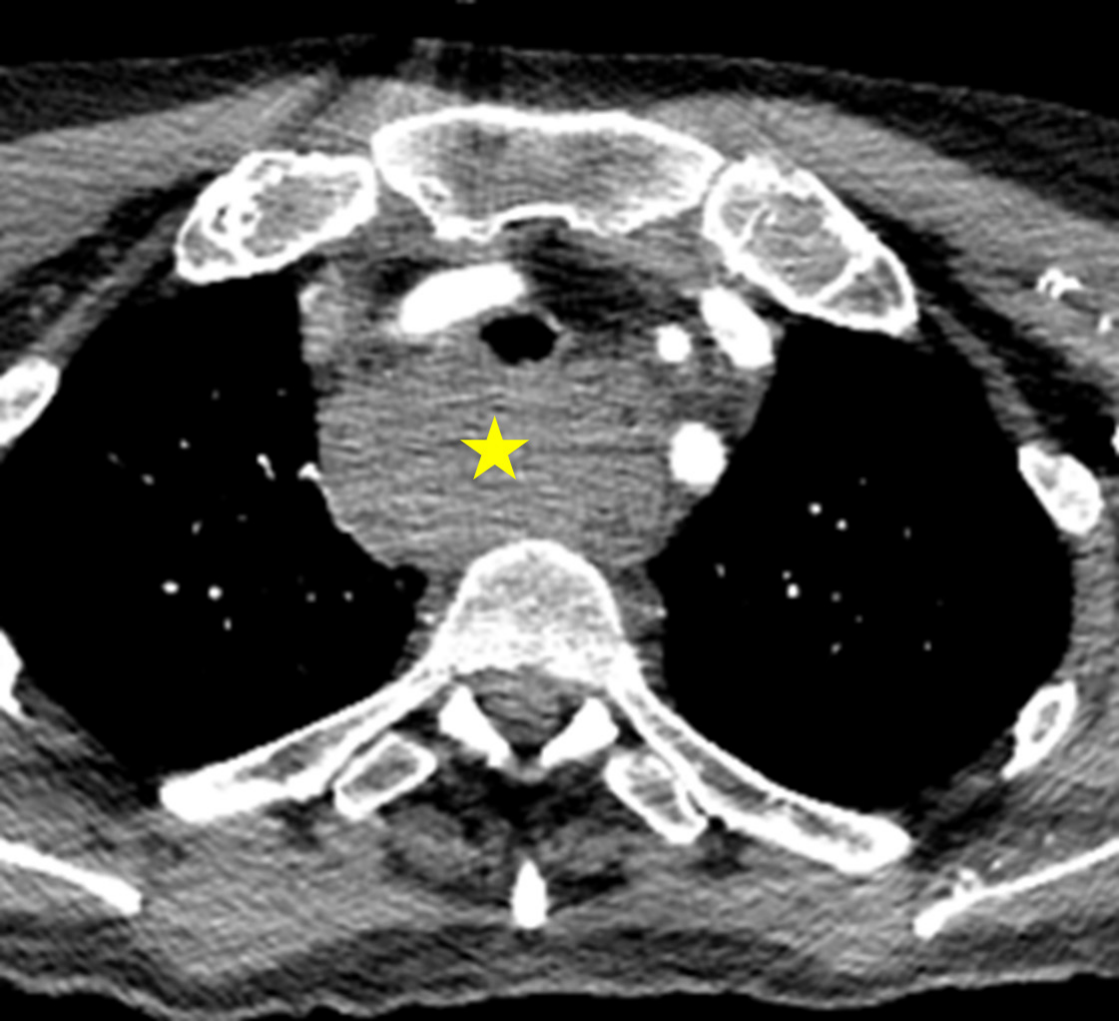
Inferior extension of the haematoma, extending down the ‘danger’ space into the mediastinum.

The CT demonstrated opacification of the entire retropharyngeal (danger) tissue space (mean 65HU), extending from the skull base to the mediastinum, with marked mass effect, resulting in effacement of the nasopharynx and supraglottic larynx anteriorly. (Figures 1 and 2)

There was no contrast extravasation and no radiographic evidence of a penetrating injury or acute fracture.

**Figure 3.**
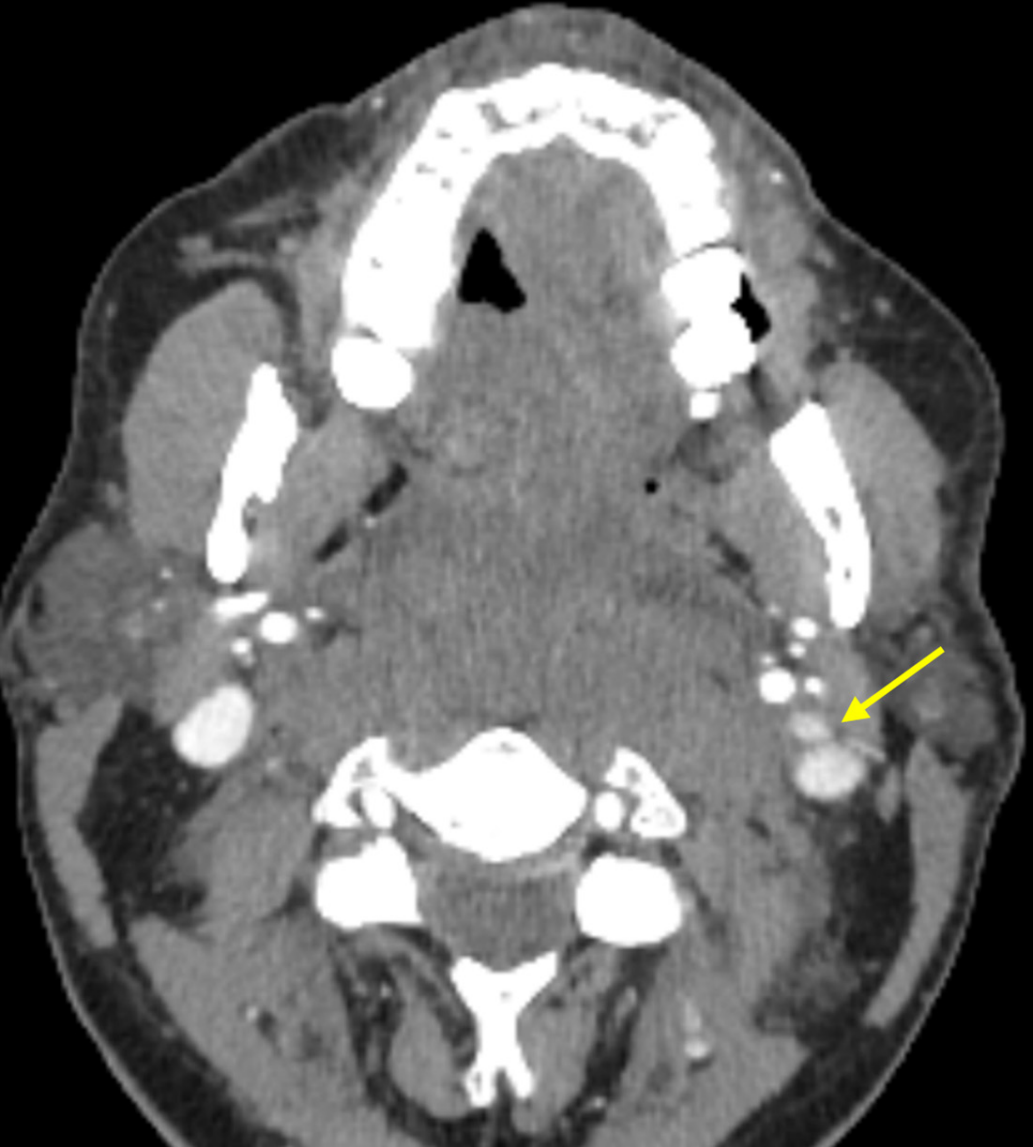
Luminal irregularity of the left IJV.

Delayed venous phase imaging was not performed, however both internal jugular veins were partially opacified in the arterial phase. Equivocal luminal irregularity was identified within the left internal jugular vein, immediately posterior to the ramus of the mandible, extending approximately 22 mm craniocaudally. This correlated to the site of pain and site of impact (Figure 3).

**Figure 4.**
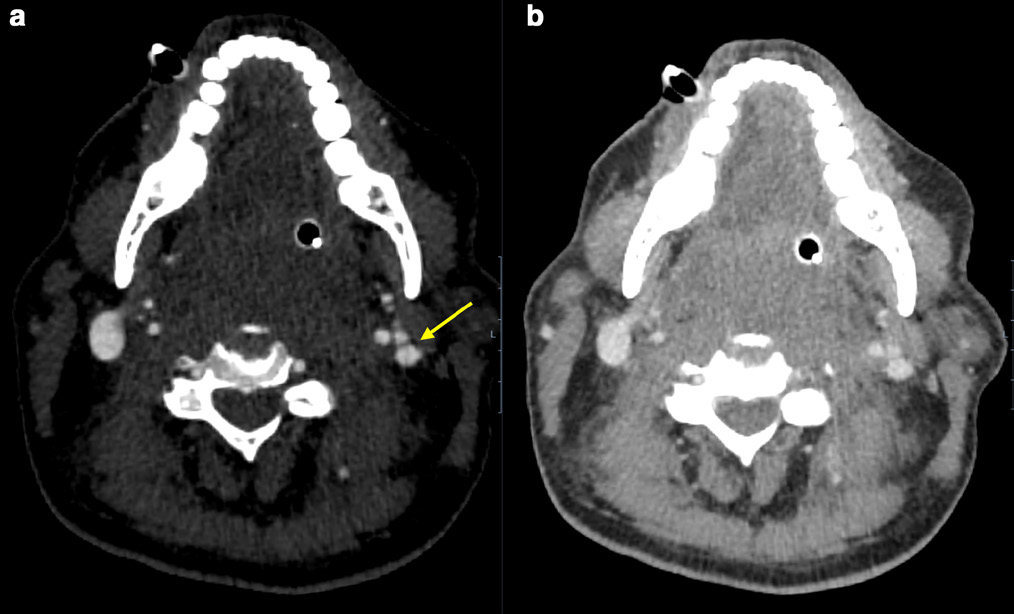
(a, b) Luminal disruption again noted at the left internal jugular vein.

Following stabilisation on the intensive therapy unit (ITU), approximately five hours after initial imaging, triple-phase (unenhanced, arterial and venous phase) contrast-enhanced CT of the neck was performed. There was change in mass effect and no contrast extravasation was identified on any phase of imaging. Significantly, however, the repeat study again demonstrated luminal irregularity within the left internal jugular vein at the site of injury, consistent with acute venous intimal disruption (Figure 4a and b).

## Differential diagnosis

The initial working diagnosis was that of arterial haemorrhage given the immediacy and extent of airway compromise. Following the initial angiographic imaging, arterial injury remained within the differential with possible tamponade due to compartmental mass effect but acute venous injury was also considered, given the focal segment of luminal irregularity in the left IJV.

## Treatment

The patient required intubation to secure the airway and mechanical ventilation for 11 days in a level III, intensive therapy, environment. No immediate or delayed surgical intervention was necessary. At day three, there was no significant improvement in swelling (objectively assessed by neck circumference measurements). Naso-endoscopic assessment was attempted but non-diagnostic due to extensive mucosal oedema. A repeat dual-phase (arterial and venous) contrast-enhanced CT of the neck demonstrated maturation of the retropharyngeal haematoma and persistent luminal injury to the left IJV. The arterial tree was intact.

**Figure 5.**
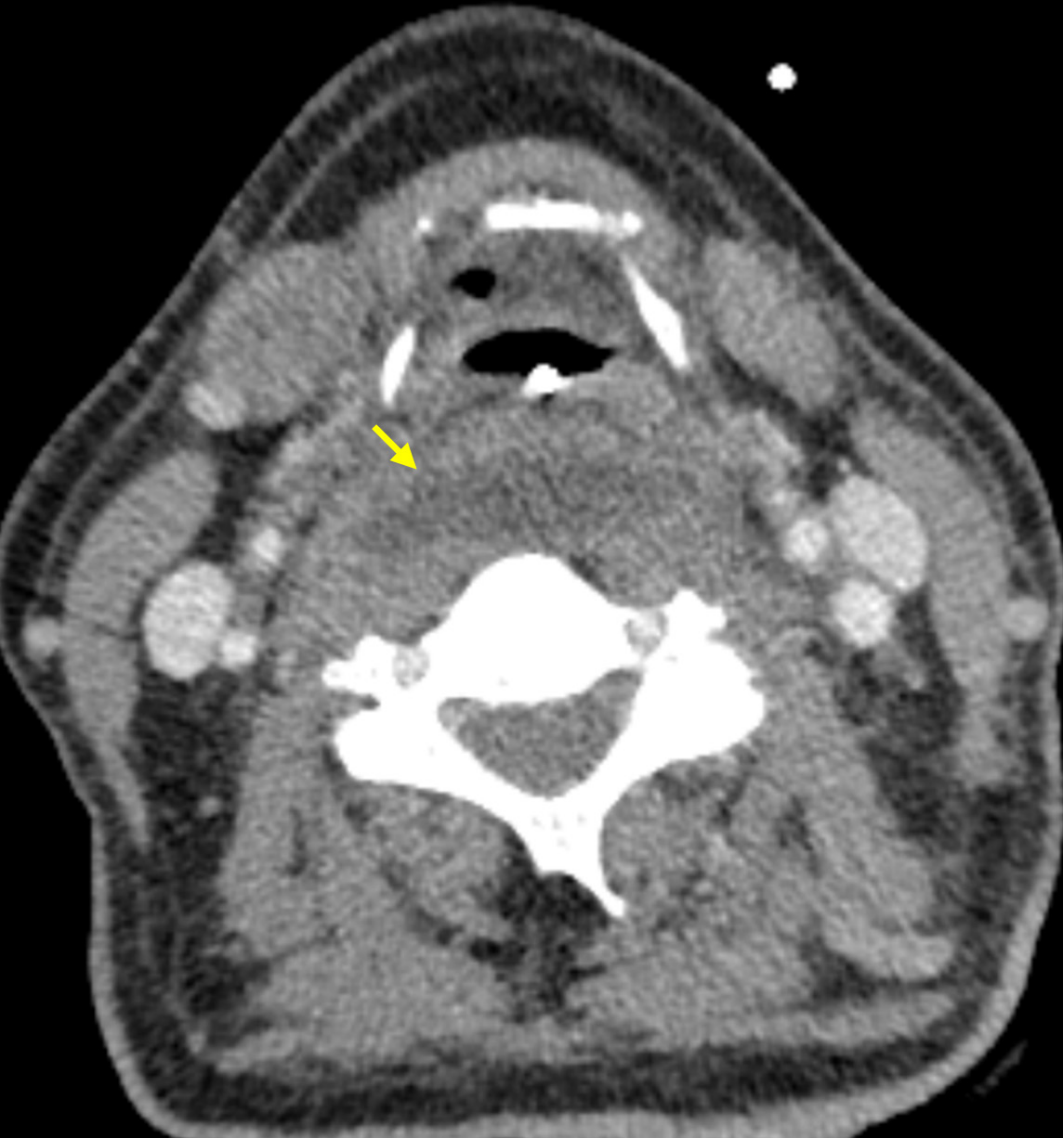
Significant reduction in size of the retropharyngeal haematoma, with new rim enhancement suggestive of infection.

At day seven, the patient became pyrexial (37.5°C), pus was observed to be discharging from his oral cavity and his CRP rose to 137 mg l^−1^ (from 6 mg l^−1^). Delayed venous phase imaging of the neck and chest demonstrated overall reduction in volume of the retropharyngeal haematoma. However, the collection demonstrated heterogeneous attenuation and focal rim enhancement consistent with infected haematoma (Figure 5). Broad spectrum antibiotics were started, as per local protocol.

At day 11, there was notable reduction of the neck swelling and the patient was successful extubated and stepped down from intensive care.

A final delayed venous phase study of the neck at day 19 demonstrated almost complete resolution of the retropharyngeal haematoma, with only a trace residual collection identified anterior to the pre-vertebral soft tissue and non-specific inflammatory stranding.

The patient was discharged at day 21 with complete resolution of symptoms.

## Outcome and follow-up

The patient was followed up in the ENT outpatient clinic two weeks following discharge home. He had made a full recovery and has been discharged from our care.

## Discussion

Acute airway compromise is a well-recognised complication of vascular injury in the neck. However, this is most commonly associated with an acute arterial injury, particularly following penetrating trauma.^1^ The onset of signs is often rapid and associated with significant morbidity and mortality.^2^

Venous injury secondary to blunt trauma in the neck is rare. Review of the literature identifies only a few cases of retropharyngeal haematoma secondary to established blunt force trauma. In both documented cases of isolated internal jugular vein injury in the absence of a penetrative mechanism of injury,^3,4^ the patients were involved in road traffic collisions. In one case, the patient’s neck was described to have been under sustained and significant pressure from the [car] wreckage.^4^ In our case, however, the cause of the injury, automated closure of *tube* doors, was a single, ‘transient’ event.

The delayed onset of initial symptoms and signs combined with the eventual rapid development of airway obstruction is unusual but explainable. The internal jugular venous system is a relatively low-pressure system with little to no elastic recoil in the intimal layers. It is, therefore, unsurprising that disruption of the venous walls may result in slower blood loss than an arterial injury would. In addition, the unique anatomical relationships of the investing layers of cervical fascia results in numerous potential spaces within the head and neck that may accommodate expansion of a haematoma without immediate clinical symptoms or signs. Therefore, the presentation of haemorrhage of venous origin may be variable rather than immediate.

The retropharyngeal tissue space, also termed the ‘*danger* space’, is a well-described ‘potential’ space that lies between the thick prevertebral and retropharyngeal fascial layers. It is bounded by the base of skull superiorly and the posterior mediastinum inferiorly, thus a large volume of fluid may extend throughout this space. Significant mass effect on the aerodigestive tract and the adjacent structures is well recognised, particularly in the context of organising haematoma.

A key diagnostic challenge in both arterial and venous bleeding is that the development of a large volume retropharyngeal haematoma results in natural tamponade as the extravascular pressure rises, compressing and often arresting the bleeding point.

The key focus of the emergency management of an acute obstructed airway in the context of a recent neck injury is establishing definitive control of the airway. Aggressive multi-disciplinary management is often required. Although a collar would unlikely to have been tolerated in these circumstances, minimal neck movement and manipulation is recommended to preserve the cervical spine alignment, reducing the risk of haematoma rupture and ischaemic spinal complications.^5^

The definitive management of significant venous injury as a result of blunt trauma remains a matter of debate in the literature. Both expectorate (conservative) or surgical intervention methods are described, predominantly depending on the clinical signs, the size and age of the haematoma. Transoral or percutaneous drainage is described as advantageous in large or infected haematomas, or where haematoma fails to resorb.^2,3,6^ However, this carries an increased risk of infection, particularly via the transoral approach.^6^

## Conclusion

Acute vascular injury must always be considered in all cases of penetrating and blunt trauma to the neck. Whilst rare, we suggest a low index of suspicion for venous injury in the context of blunt trauma. Thus, when protocolling acute imaging investigations, we advocate the use of triple phase CT angiography (unenhanced, arterial and venous phase) as the standard of care in this patient cohort, despite the relative infrequency of venous injuries.

## Learning points

- In the context of blunt force trauma of the neck, venous injury should be considered alongside arterial injury.
- Consider triple phase angiographic imaging in blunt force trauma to the neck, especially where there are acute upper aerodigestive symptoms or signs.
- Have low threshold for rescanning patients who have sustained blunt force trauma of the neck where the clinical picture is at odds with an apparently normal initial study.

## References

[1] Simmons JD, Ahmed N, Donnellan KA, Schmieg RE, Porter JM, Mitchell ME. Management of traumatic vascular injuries to the neck: a 7-year experience at a level I trauma center. Am Surg 2012; 78: 335–8. doi: 10.1177/000313481207800343 22524773

[2] Daniello NJ, Goldstein SI. Retropharyngeal hematoma secondary to minor blunt head and neck trauma. Ear Nose Throat J 1994; 73: 41–3. doi: 10.1177/014556139407300110 8162871

[3] Park CY, Kang WS, Seo SH, Moon SN. Nonsurgical management of traumatic internal jugular vein rupture using direct compression. Trauma Image Proced 2018; 3: 62–4. doi: 10.24184/tip.2018.3.2.62

[4] Clark JD. Blunt neck trauma: torn internal jugular vein after a motor vehicle crash. J Emerg Nurs 2008; 34: 249–50. doi: 10.1016/j.jen.2008.02.022 18558264

[5] Lazott LW, Ponzo JA, Puana RB, Artz KS, Ciceri DP, Culp WC. Severe upper airway obstruction due to delayed retropharyngeal hematoma formation following blunt cervical trauma. BMC Anesthesiol 2007; 7: 2. doi: 10.1186/1471-2253-7-2 17352800 PMC1828150

[6] Park JH, Jeong E-K, Kang D-H, Jeon SR. Surgical treatment of a life-threatening large retropharyngeal hematoma after minor trauma: two case reports and a literature review. J Korean Neurosurg Soc 2015; 58: 304. doi: 10.3340/jkns.2015.58.3.304 26539280 PMC4630368

